# The Sufferings of Christ

**Published:** 1888-03-10

**Authors:** 


### Words of Consolation.

[Communications for this column should be addressed "Chaplain," care of the Editor of The Hospital, who will gratefully welcome any suggestions or inquiries from matrons, nurses, and the staff generally.]

### LXIX.—The Sufferings of Christ.

*For Reading to the Sick.*

The utter rejection of our blessed Lord's humanity by the creatures of His own making may also afford some clue towards the solution of a difficulty which some of us cannot help feeling about lost souls, more especially such as have once been loved or cared for. In the case of any whom we have loved being lost, how could we be happy even in Heaven knowing this. We cannot entertain the fear of their being lost now without pain. Could we ever endure the fact? We must remember now that, even at their worst, they are not lost yet. Why not? Because, however degraded or depraved, God's image is not completely effaced from their being. There is still in them something, it may be very little, but still something to appreciate or to care for—something they once possessed in the way of natural gifts or disposition which we cannot help liking, though it may be obvious that the grace of God has been persistently rejecte d. But when even this some thing has been taken away, this semblance of good, or this natural talent which was merely kept, but not improved; where "even that which he hath is taken away," and there is nothing left in the forsaken sinner, but what is of sin and therefore hateful to God: will there be anything for us to care for *then?*

Probably not. The wretched worm, the outcast, the ruined soul, stripped of all that was lovable or gracious, even bereft of the little good which it *seemed* to have, will then have nothing left about it to love. The soul which hath despised and rejected the love of Jesus Christ crucified, trodden under foot the Son of God, and put Him to an open shame, puts itself at last outside even the range of God's love.

What should we care for a home once dear to us if after a lapse of years we came back and found it utterly desolate, deserted, stripped of every association which once made it our home? Imagine the garden a wilderness, the furniture, the pictures, all gone, the house a ruin—or worse—given over to reptiles and corruption. Surely the place thereof would know us no more. We would rather not identify the spot. We might linger awhile amid the chaos, if perchance we could discover a single relic of any kind that made the past dear to us. But finding none, could we rest there? No. Should we not cry out: "What is there left to keep us here? We can see nothing but a refuge for worms and loathsome creatures. All that once made this our home has been taken away; and with the 'all' our love must be translated as well."

If instead of a home ruined on earth, we substitute a person we once knew; and suppose that person ruined, deprived of every quality of grace, of every feature in character which could have borne a resemblance of good, what will there be left to attract our affections any more? What will there be found remaining to love? Very dimly in the great darkness of Calvary the rejection and desolation of Christ's humanity seem to emphasise the sentence of the Judge, "From him that hath not shall be taken away even that which he seemeth to have." (Luke viii. 18.)

